# Type 2 Innate Lymphoid Cells: Protectors in Type 2 Diabetes

**DOI:** 10.3389/fimmu.2021.727008

**Published:** 2021-08-19

**Authors:** Jacob D. Painter, Omid Akbari

**Affiliations:** Department of Molecular Microbiology and Immunology, Keck School of Medicine, University of Southern California, Los Angeles, CA, United States

**Keywords:** ILC2, type 2 diabetes, neuroimmunology, immunometabolism, thermogenesis, immune regulation

## Abstract

Type 2 innate lymphoid cells (ILC2) are the innate counterparts of Th2 cells and are critically involved in the maintenance of homeostasis in a variety of tissues. Instead of expressing specific antigen receptors, ILC2s respond to external stimuli such as alarmins released from damage. These cells help control the delicate balance of inflammation in adipose tissue, which is a determinant of metabolic outcome. ILC2s play a key role in the pathogenesis of type 2 diabetes mellitus (T2DM) through their protective effects on tissue homeostasis. A variety of crosstalk takes place between resident adipose cells and ILC2s, with each interaction playing a key role in controlling this balance. ILC2 effector function is associated with increased browning of adipose tissue and an anti-inflammatory immune profile. Trafficking and maintenance of ILC2 populations are critical for tissue homeostasis. The metabolic environment and energy source significantly affect the number and function of ILC2s in addition to affecting their interactions with resident cell types. How ILC2s react to changes in the metabolic environment is a clear determinant of the severity of disease. Treating sources of metabolic instability *via* critical immune cells provides a clear avenue for modulation of systemic homeostasis and new treatments of T2DM.

## Introduction

Type 2 diabetes mellitus (T2DM) is a disease characterized by the inability to maintain healthy glucose levels due to systemic insulin resistance. There is complex immunology involved in the pathogenesis of T2DM which makes it an incredibly challenging disease to treat. Patient heterogeneity caused by a mix of environmental and genetic factors leads to a wide range of phenotypes observed in the clinic (1, 2). These contribute to immunological changes which play a part in the pathogenesis of T2DM. Understanding the immunology of tissues involved in T2DM and their contribution to causing or preventing its pathogenesis is critical for opening up new avenues to treatment. Modern treatments of T2DM include diabetic medication and insulin supplementation for regulation of blood glucose levels accompanied by lifestyle changes such as exercise and improved diet. However, these treatments fail in treating the root cause of the disease. Changes in the landscape of cells in effector tissues of diabetes are going to be critical in future treatment options. Immune cells provide a clear and present target for amelioration of insulin resistance, maintaining tissue homeostasis, and diabetes as a whole (3). Insulin resistance is a hallmark of T2DM, and immune cells have a key role in its development through the perpetuation of inflammation in diseased tissues (4). Regulation of immune cell populations can ameliorate tissue inflammation and the causes of insulin resistance and T2DM.

Due to the heterogeneity of the human population, determining the root causes of metabolic instability and insulin resistance in patients is challenging, making it a major subject of interest (5, 6). A combination of insufficient β-cell-derived insulin and peripheral tissue insulin resistance both play a role in the development of the disease. How these two pathogenic characteristics develop is a major point of interest. Evidence points to inflammation caused by a dysregulated innate immune system as a major player these processes (4, 7). Innate immune cells produce the first immunological response to pathogens and express a variety of pattern recognition receptors (PRRs) able to recognize pathogen-associated molecular patterns (PAMPs) which subsequently activate downstream inflammation (8). Cells involved in this process include neutrophils, eosinophils, natural killer (NK) cells, monocytes, macrophages, and dendritic cells (DC). In obesity-induced T2DM, chemotactic signaling originating from inflamed adipose, liver, and muscle tissues leads to innate cell infiltration, further inflammation, and ultimately insulin resistance (9). Previously ignored in the pathogenesis of many disorders until recently, type 2 innate lymphoid cells (ILC2s) have recently come into interest for a variety of allergic and metabolic diseases. ILC2s, unlike their adaptive T helper type 2 (Th2) cell counterparts, do not express antigen receptors, however, they are activated by molecular signals such as interleukin(IL)-25, IL-33, and thymic stromal lymphopoietin (TSLP), and lipid mediators (10). Unlike T cells, innate lymphoid cells are deposited into tissues early during ontogeny and are integral in the regulation of each organ and its functional changes due to the environment (11, 12). They also have been observed to traffic between organs and inflamed tissues (13). ILC2s have been found to regulate homeostasis in a variety of tissues and to be the first responders to tissue damage such as that found in adipose, gut, skin, and lungs (14).

Adipose tissue makes up one of the largest reservoirs of cells in the body and is quickly becoming a point of interest in disease due to its large quantity of immune cells. Fat deposits are broken into different subsets as white (WAT), brown (BAT), or beige fat; in addition, there are different subsets of white fat such as visceral adipose tissue (VAT), found in the abdominal area, and subcutaneous found below the skin. VAT is one of the most important tissues involved in T2DM due to its association with the disease in both obese (15) and non-obese (16) patients. In stark contrast, BAT contains more active mitochondria and is involved in thermogenesis and generation of heat from fat storage; BAT is equipped for healthy energy expenditure and prevention of T2DM (17). BAT is distinct in location and function providing thermal energy instead of energy storage. Therapeutic targeting of brown fat and initiating conversion of WAT to BAT to an intermediate beige state provides a promising treatment option for patients with excessive calorie intake or obesity-induced T2DM. ILC2s reside in WAT of both lean mice and humans (18, 19), and have a clear and present role in maintaining homeostasis in these metabolically active adipose tissues through the promotion of VAT to BAT-like adipose tissue, in a process referred to as beiging (19–21). Beige adipose tissue is critical for the healthy function of adipose tissues and is inducible in WAT (22). Most important in this process of adipose tissues is uncoupling protein-1 (ucp1) coded by the gene *UCP1*. Ucp1 protein short circuits the mitochondrial membrane electrochemical gradient, which results in both less ATP production and the release of energy in the form of thermal heat (23). Interestingly, there are slight differences between beige and brown adipocytes that can be distinguished and they are considered as individual populations (24). Generally, brown adipocytes have high ucp1 concentrations at basal conditions, while beige adipocytes express it in response to β-adrenergic receptor agonists and peroxisome proliferator-activated receptor-γ (PPARγ) signaling (25). PPARγ is an essential factor in driving lipid metabolism through droplet formation and fatty acid uptake in adipose tissue (26). PR domain zinc finger protein 16 (prdm16), a transcription factor expressed in the BAT relative to WAT, is also responsible for thermogenic processes, beige adipocyte development, and improvements in glucose tolerance in mice studies (27). RNA sequencing of VAT lysates has shown increased gene expression of *PRDM16*, *PPARγ*, and *UCP1* in response to specific activation of ILC2s (20, 28), showing that they have a key role in the development of beige adipocytes.

The key immunological hallmark associated with dysfunctional metabolic homeostasis is the recruitment and expansion of inflammatory macrophages (M1) in the adipose tissue. Inflammation caused by damaged adipocytes initiates a cascade of damage signals leading to severe inflammation. In response, M1 produce damaging inflammatory signals leading to deficiency of protective cell types essential for proper tissue maintenance and homeostasis (29). In cases of obesity, VAT becomes a large immunological reservoir for the production of inflammatory signals which impact systemic metabolism. ILC2s are impacted severely due to this inflammatory environment and their numbers sharply decline in cases of obesity (18, 19). While classically activated M1 macrophages are stimulated by bacteria stimuli *via* PPRs, adipose macrophages respond to changes in metabolic stimuli which complicate the classical M1/M2 axis (30). Changes in glucose saturated fatty acids, and insulin and upregulate pro-inflammatory cytokines IL-1β and TNF-α are most commonly associated with T2DM (30). Hypertrophic adipocytes in cases of obesity perpetuate the inflammation and concentrations of lipid species *via* adipose tissue macrophages (ATM). There are distinct populations of ATMs that are found in the obese fat tissue of mice and humans; murine Ly6c marks adipogenic ATMs, while CD9 marks proinflammatory ATMs in both mice and humans (31). During obesity, interferon regulator factor 5 (IRF5) regulates the pro-inflammatory polarization of ATM, and deficiency of IRF5 promotes a type 2 immune regulatory environment *via* ATM-derived transforming growth factor β (TGF-β) (32). Elimination of this inflammatory reservoir is key in T2DM treatments, where activated ILC2s can initiate a more immunoregulatory environment.

ILC2s control homeostasis through regulation of other innate cell populations and ultimately adipocyte gene expression which makes them a clear target for T2DM treatments. ILC2s reside in white adipose tissue of both lean mice and humans (18, 19), and there they act as a direct source of methionine-enkephalin that directly stimulate the activity of beige adipose tissue (19). VAT ILC2s suppress harmful inflammation found in T2DM through the production of type two cytokines such as IL-5 and IL-13, which stimulate eosinophils production of IL-4 and differentiation of macrophages to anti-inflammatory state (identified as alternatively activated macrophages, AAMs, M2) in white adipose tissues (18). With the adoptive transfer of these ILC2s, adiposity and glucose tolerance were also improved in obese mice showing the therapeutic potential of activation of these cell types might have (33). It is also important to note that in obese patients, ~40% of the resident cells in adipose tissues are macrophages (34), making M2 polarization of these cells crucial in the regulation of immunological balance and metabolism of adipose. ILC2s can directly regulate metabolic homeostasis by increasing the expression of *UCP1* in adipocyte populations (19). Pancreatic islet ILC2s also activate DCs for the release of retinoic acid (RA) to promote insulin secretion (35). ILC2s secretion of type 2 cytokines are important in the landscape of metabolic homeostasis and downstream insulin signaling as their primary effector functions seek to avoid type 1 immunity, a key player in metabolic dysfunction (36).

While all ILCs are IL-7R+, these cells are further broken up into three subsets each characterized by transcription factor expression and cytokine production that vaguely mirror classical T helper subsets, ILC2s are defined by the transcription factor GATA-3, while type 1 innate lymphoid cells (ILC1) are defined by T-box transcription factor TBX21 (T-bet) and type 3 innate lymphoid (ILC3) cells are defined by retinoic acid receptor-related orphan receptor gamma T (RORγt). Defined transcription factors also facilitate further defined cytokine production by each subset. Classical ILC2s produce IL-5 and IL-13, while ILC1s produce interferon-gamma (IFN-γ) and TNF, and ILC3s produce IL-17A and IL-22. In addition to single transcription factor expression, additional combinations may also be introduced such as ILC3s with co-expression of RORγt and T-bet (37). Additionally, this co-expression is governed by the transcription factor c-MAF; these cells are able to produce both IL-22 and IFN-γ. Significant plasticity has been observed in ILC populations, however, the specific role of plasticity and adipose ILC2s has yet to be fully described (38). Evidence points to ILC2s being able to adapt to the cytokine milieu. High concentrations of inflammatory cytokines present in cases of T2DM would have a great impact on tissue-resident and circulating ILC2 populations contributing to disease. How trained ILC2s and ILC2 committed progenitors respond to these stimuli has yet to be fully discovered.

Currently, there is a gap in knowledge between what we understand about the tissue-specific immune system and how we can prevent and treat diabetes. Key immune cellular mediators, such as ILC2s, are essential for the maintenance of homeostasis in adipose tissues. In this review, we will be discussing the role of ILC2s in the immunology behind the pathogenesis of T2DM. ILC2 numbers steeply decline in response to obesity in both murine and human studies (18, 19). Understanding how this happens due to interaction with the cytokine milieu, adipose stromal cells, and other immune cells will be discussed for implications into metabolic homeostasis and insulin resistance contributing to disease. Trafficking and maintenance of immune cells from the bone marrow to adipose tissues is also necessary as it contributes to a deficiency in type 2 response and an exacerbation of inflammation in diabetic active tissues. Immunotherapeutic strategies targeting ILC2s leading to their survival and expansion during cases of obesity provide a clear and present treatment strategy for T2DM. There is also a necessity for cell-specific agents able to activate individual cell subsets which would allow for treatments of individual phenotypes. With ILC2s ability to impact both the beiging of WAT and downregulate inflammation in adipose tissues, they provide a specific target for future T2DM treatments. With clustering patients by immunological differences, regulation of the immune system through specific immunotherapies provides a clear avenue for diabetes treatment.

## ILC2s in Diabetic Active Tissues

In addition to the adipose tissue, ILC2s are also present in the liver. Hepatic ILC2s maintain a small naïve inactive phenotype in healthy liver but become highly activated by IL-33 in cases of hepatitis (39). In addition, IL-33-activated hepatic ILC2s seem to show resistance to both IFN-γ and plasticity induced by IL-12 (39). Hepatic ILC2s have been shown to be players in pathologic tissue remodeling and fibrosis (40, 41). In human studies, intrahepatic ILC2s secreted amphiregulin and IL-13 to drive regeneration and proliferation of bile ducts through EGFR activation; they exerted both pro-fibrotic effects and protective responses in end-stage liver disease (42). Stressed hepatocytes secrete large quantities of IL-33 in disease which is responsible for activation of ILC2s. Liver fibrosis is directly relevant to T2DM as one in six patients with T2DM display moderate-to-advanced fibrosis of the liver (43). It is unlikely that ILC2 activation is the root cause of liver fibrosis as they are reactors to IL-33 which is released due to liver damage. In addition, hepatic stellate cells are the primary producers of matrix proteins in the liver and are highly activated by IL-33 (44). ILC2s likely synergize with hepatic stellate cells to promote hepatic fibrogenesis in cases of stress to initiate repair, but in advanced cases of liver damage, fibrosis exacerbates the state of disease likely contributing to systemic metabolic dysfunction. ILC2s also contribute to stellate cell activation by IL-13 secretion (41). Interestingly, acute models of liver inflammation observed a protective effect of IL-33 on hepatocytes and liver function, data suggests that IL-33 works as a protective mechanism in acute liver damage but stimulates tissue fibrosis in chronic injuries (45). A line is likely crossed in case of chronic liver injury in which ILC2s contribute to disease. In acute liver damage, they initiate repair and normal liver function. A patient’s level of liver inflammation is a determinant of how ILC2s function in fibrosis and contribute to T2DM. Little is known about the role of hepatic ILC2s in the pathogenesis of diabetes, but inflammatory environment likely impacts the role they play.

In the pancreas, ILC2s contribute to the protection of islet β-cells responsible for insulin production. In cases of T2DM, pancreatic-islet inflammation leads to failure of the pancreas to secret insulin. Pancreatic ILC2s respond to IL-33 produced by resident islet mesenchymal cells (35). In turn, activated ILC2s secreted IL-13 and colony-stimulating factor 2 (CSF2) which induced retinoic acid production by macrophage and dendritic cells (35). RA signaling cascade resulted in increased β-cell insulin secretion (35). Interestingly, conditions of T2DM such as chronic high concentrations of glucose, IL-1β, and palmitate increase IL-33 production by mesenchymal cells and disrupt the IL-33/ILC2 axis leading to less insulin secretion (35). IL-33 injection rescued islet function and secretion of insulin *via* ILC2s (35). Also, tissue damage due to acute pancreatic β-cell failure led to increased IL-33 and ILC2 numbers (35). Promoting survival of ILC2s in pancreatic islets in cases of T2DM would aid insulin secretion and maintain healthy glucose levels.

## ILC2s Increase Systemic Metabolism

Browning of the adipose is a complex process of signals leading to increased energy expenditure in the form of heat. These beige adipocytes have substantially increased metabolism and mitochondrial activity. ILC2s play a major role in the protection of thermogenic processes through protection against type 1 inflammatory response which impairs their function (46). In multiple models, ILC2 specific activation increases the expression of a variety of thermogenic genes associated with adipose tissue homeostasis and glucose tolerance. These genes include *UCP1*, *PRDM16*, peroxisome proliferator-activated receptor-γ coactivator 1 alpha (*PGC-1α*), and *PPARγ* (20, 28). ILC2s secrete methionine enkephalin which directly stimulates the formation of beige adipocytes (19). In addition to beiging, activation of ILC2s has been shown to increase the expression of genes associated with higher metabolism including genes for mitochondrial chain complexes I (*Nd1* and *Nd2*), III (*Cytb*), IV (*Cox1a*), V (*Atp6*), and tyrosine hydroxylase in VAT lysates (28). Their maintenance of homeostasis and healthy tissue growth in the presence of a high-fat diet (HFD) and obesity is mediated by their support of anti-inflammatory ATM populations (18, 19, 47, 48). Recently, ILC2s have been observed to regulate diet-induced obesity and chronic inflammation *via* control of saturated fatty acid absorption in VAT (46). ILC2s are central regulators of adipose homeostasis and work directly and indirectly with resident adipose cells to maintain healthy tissue function. This new data also suggests that saturated fatty acid induce apoptosis of murine macrophages in addition to apoptosis of adipocytes. Injection of IL-33-activated ILC2s decreased concentration of saturated fatty acids restoring glucose tolerance and fatty acid metabolism in IL-33 receptor knock-out mice (46). Proper adipose tissue function is a key determinant in the pathogenesis of T2DM due to its large scope in metabolism.

## Immune Cells and ILC2s in T2DM

Regulation of metabolic balance by the immune system is key in protection against metabolic disturbances and T2DM. Obesity increases the release of proinflammatory cytokines and metabolites which lead to the development of T2DM. The release of TNF-α (9), 1L-1β (49), and IL-6 (50) directly lead to downstream effectors that seek to not only activate the inflammasome through NF-κB activation in resident cells but also regulate the effects of insulin signaling. Through the suppression of insulin receptor substrate, TNF-α ultimately leads to suppression of PI3K and AKT signaling needed for proper glucose response (9). Chronic release of the cytokine in cases of obesity and other conditions leads to effects in adipose tissue, pancreas, liver, and muscle (51). Beyond their direct effects on systemic homeostasis, how ILC2s fight harmful inflammation in a variety of contexts will be described here as their direct regulation of a changing adipose environment is key in the prevention of T2DM and possible future treatments. It is crucial to look at both the cytokines and cell-to-cell interactions involving ILC2s and other resident cells to understand the impact on metabolic disease.

There are a variety of immune cells involved in the pathogenesis of T2DM. Their overall effect on the pathogenesis of the disease is highly dependent on their location and ability to traffic to inflamed tissues (52, 53). The most recognized cause of T2DM is obesity and weight gain (54). During the onset of obesity, the trafficking of immune cells within adipose tissues is disrupted (55, 56). Control over infiltration of immune cells is poorly understood especially in the case of T2DM, however, CD11c^+^ cells are involved in the regulation of lymphocyte trafficking to diabetic islets in type 1 diabetes (T1D) (57). The interplay between all cell types, cytokines, and tissues is key in identifying how T2DM develops. VAT adipocytes, unlike beneficial subcutaneous WAT adipocytes (58, 59), display more lipolysis and susceptibility to apoptosis (60, 61). Apoptosis from adipocytes causes the release of pro-inflammatory adipokines associated with systemic insulin resistance and T2DM (62). Therefore, treatments that control VAT homeostasis are essential for the prevention of sustained inflammation. VAT-produced free fatty acids (FFAs) can travel directly to the liver *via* hepatic blood supply which makes them a key player in the regulation of liver metabolism and hepatic insulin resistance (63). BAT is important in the conversion of fat to energy in the form of heat and is also responsible for fighting metabolic imbalances such as those found in T2DM (17). Maintaining the balance between beige adipose tissue and WAT is essential to controlling the onset of metabolic syndromes. Adipocyte genes such as Hoxa5 (64) and Hoxc8 (65) are some of the many crucial genes that play a role in the beiging of WAT to maintain healthy energy expenditure. Ucp1 is the most common protein studied in the browning of WAT adipocytes, programming them for increased caloric expenditure (22, 66). ILC2s have been shown to directly increase *UCP1* expression and protein levels in adipocytes through secretions of methionine-enkephalin peptides and cytokines, showing their regulatory control over adipose homeostasis (19–21). Overall, maintaining homeostasis in VAT is essential to preventing systemic metabolic syndrome and insulin resistance.

### Macrophage Polarization in the Adipose

ILC2s can indirectly regulate type 1 cytokines such as TNF-α and IL-1β, found in cases of obesity, through influencing M1 macrophage polarization. TNF-α has a significant effect on the insulin signaling pathways and directly contributes to insulin resistance and T2DM. It impairs insulin signaling through serine phosphorylation of insulin receptor substrate 1 (IRS-1) and reduction of glucose transporter type 4 (GLUT-4) expression (67). This reduction in GLUT-4 expression decreases glucose entry into cells and contributes to a lack of glucose homeostasis. The modification of IRS-1 leads to its change to an insulin receptor inhibitor in murine adipocytes, therefore abrogating the effects of insulin in adipose tissues (68). IL-1β suppresses insulin-induced glucose transport, lipogenesis, and IRS-1 phosphorylation in human and murine cell lines (69, 70). Blocking of IL-1β reduces inflammation and hyperglycemia in mouse models (71). ILC2s’ ability to polarize M2 macrophages away from an M1 phenotype would affect the production of TNF-α and IL-1β making them helpful in the treatment of T2DM. A TNF-α neutralizing antibody has also been used to ameliorate TNF-α induced insulin resistance in adipocytes (72). Cellular modulation of TNF-α and IL-1β expression through ILC2 activation and promotion of M2 macrophages may provide a longer-term treatment for T2DM.

The polarization of macrophages between M1 and M2 is the critical player in healthy adipose tissue. M2-like ATMs regulate many of the healthy functions of adipose tissue such as tissue growth and inflammation (73). While depletion of CD206+ M2-like macrophages has been shown to increase glucose metabolism in lean and obese mice (74), they function in a key niche in maintaining beige and white adipocyte progenitor populations and priming adipose tissue for healthy adipose expansion in case of high nutritional intake (73). The depletion of M2-like macrophages promoted adipogenesis responsible for observed improvements in glucose metabolism, which explained the study’s seemingly contradictory results compared to other publications showing M2-like macrophages promoting healthy metabolism (19, 47, 48). Secretion of cytokines IL-5 and IL-13, by ILC2s, regulate the generation of M2 macrophages which promote tissue homeostasis in VAT (18). IL-5 contributes to eosinophil accumulation and production of IL-4 which further aids macrophage differentiation. In cases of adipose inflammation and subsequent insulin resistance, generation of these AAMs by adoptively transferred ILC2s contributed to less adiposity and glucose tolerance in obese mice; in contrast, *in vitro* expanded Th2 cells were shown to not have these same effects when transferred (33). This shows that ILC2 activation is more beneficial in the regulation of adiposity and glucose homeostasis than adaptive T cells. Likewise, ILC2s are also affected by macrophages. M1 macrophages can suppress ILC2 activity through the secretion of type 2 interferon, IFN-γ, which suppresses their proliferation and secretion of cytokines (75, 76). IFN-γ deficient mice fed a HFD had smaller adipocytes, improved insulin sensitivity, and AAM shift in the VAT (77). Therefore, in this context it is very possible VAT ILC2 would have played a role in improved insulin sensitivity and M2 macrophage polarization. Controlling the balance of M1 and M2 macrophage differentiation through ILC2 cytokine secretion is crucial in the maintenance of healthy adipocytes and metabolic homeostasis.

M2 macrophages, generated by ILC2 cytokine production, can regulate a variety of homeostatic processes in the adipose tissues. M2 macrophages rely on the presence of IL-13 and IL-4 for their differentiation from macrophage progenitors (78); both cytokines are either directly or indirectly produced through ILC2s. How these M2 macrophages can induce the browning of adipocytes is hotly debated (79). Activated M2 macrophages secrete many factors which upregulate WAT beige adipocyte activity and gene expression. They secrete metabolites (80, 81), growth factors (82), and cytokines (47) which promote beige adipocytes. However, the contribution and overall effect of these factors in the grand scheme of adipose tissue homeostasis remains to be determined. Multiple papers cite the induction of beige adipocytes to be from M2 macrophage produced catecholamines (18, 19, 21, 47, 48). However, others have disputed this claim, by stating M2 macrophages do not contribute to catecholamine production (83). Future perspectives will likely determine the role of catecholamines and M2 macrophages, however, it seems that M2 macrophages affect catecholamine levels through interaction with resident nerves (84) and transport of it from peripheral locations (85). The main effector in M2 macrophage’s ability to control adipose inflammation is interleukin-10 (IL-10) and interleukin-1 receptor antagonist (IL-1Ra) (86). IL-10 seeks to downregulate immune inflammation in the adipose to achieve homeostasis through prevention of IL-6 and lipid-induced insulin resistance as observed through *in vivo* administration of the cytokine (87). Another major secreted factor of M2 macrophages is TGF-β. The ability of these M2 secreted factors to affect homeostatic outcomes in the adipose depends on the developmental stages of adipocytes and the immune landscape. Pre-conditioned adipose would affect the outcome of M2 macrophage-mediated effects on a case-to-case basis in the clinic.

### ILC2s and Other Innate Actors in T2DM

Eosinophils, recruited by ILC2 secreted IL-5, can regulate adipose tissue inflammation. Like adoptive transfer of ILC2s into obese mice (20), adoptive transfer of eosinophils dampens systemic inflammation and improves WAT function lost with aging (88). Dysfunction of adipose tissue associated with old age mirrors that of remodeled adipose in cases of obesity (89). Systemic inflammation caused by dysfunctional adipose tissues plays a clear role in cases of T2DM. Eosinophils, the primary producer of IL-4 in WAT, have been associated with healthier glucose homeostasis (90). Their recruitment to WAT is largely regulated by IL-5 by ILC2s (18, 19). ILC2s are the major source of IL-5 compared to other lineage cells such as B, T, and NK cells, and depletion of ILC2s displays a significant reduction in adipose tissue eosinophils, not observed in the spleen or bone marrow (18). Interestingly, eosinophil-deficient mice showed reduced weight gain and body fat, however, they showed a more glucose intolerance phenotype than the wild type (91). Eosinophils play a key role in the homeostasis of adipose and proper cell function, and the inability to expand adipose during HFD underlies insulin resistance. Lipid storage then is diverted elsewhere, such as the liver, which can further contribute to glucose intolerance. Consequently, adipose eosinophils play a role in M2 macrophage differentiation, through IL-4 secretion, and subsequent production of IL-10 and TGF-β along with directly affecting adipocyte development. In the same way that eosinophils would not be active in aging adipose (89), poor trafficking of eosinophils to the adipose through dysfunctional or inactive ILC2s may play a role in the pathology of T2DM.

ILC2s have the potential to dampen inflammatory responses by mast cells through their production of cytokines. Human mast cells are suggested to be involved in the beiging process in adipose tissues (92). However, mast cells are also able to produce IL-6 and TNF-α (93) which are key in the recruitment of inflammatory macrophages and type 1 response. ILC2s have been shown to regulate mast cell activation through IL-13 secretion in dermal tissues. Although harmful in skin tissues, eosinophil infiltration following IL-5 secretion of ILC2s in the skin after the suppression of mast cells (93) may be beneficial in the adipose through the promotion of M2 macrophages by eosinophil-produced IL-4. Therefore, ILC2s may be able to simultaneously downregulate M1 macrophage inflammation mediated by mast cell type 1 cytokines and promote eosinophil infiltration and M2 macrophage polarization. Mast cells also produce prostaglandin D_2_ (PGD_2_) and induce chemotaxis of ILC2s through chemoattractant receptor-homologous molecule expressed on Th2 cells (CRTH2) (94, 95). Mast cells regulate ILC2 activation through the release of non-caspase proteases chymase and tryptase which cleave the activation domain of IL-33 into a more mature and potent activator of ILC2s (96). If mast cells hold an adipose tissue-specific phenotype that promotes ILC2s and adipose tissue beiging, they would be critical players in T2DM defense. Crosstalk between mast cells and ILC2s needs to be investigated fully to understand their roles in homeostasis and glucose tolerance.

### ILC2s Aid Tregs in Dampening Adipose Inflammation

There is a dynamic interaction between ILC2s and Tregs in a variety of inflammatory tissues. Tregs have long been identified for their important roles in the maintenance of VAT homeostasis and prevention of insulin resistance and glucose intolerance (97). Production of IL-33 in adipose tissue promotes ILC2 function as well as maintains Treg populations (98). Interestingly, normal expansion of Tregs was also partially dependent on ILC2s ability to express inducible T cell costimulator ligand (ICOS-L) for stimulation of ICOS+ Tregs in the adipose tissue; Tregs and ILC2s also colocalized in similar regions within the VAT. ICOS-L expression on ILC2s, and also Th2 cells, is especially high in the VAT compared to other cell types (98). In obesity-induced inflammation, sST2, a soluble isoform of IL-33R, leads to adipose ILC2 and Treg depletion ultimately leading to insulin resistance (99). It was also found that sST2 expression is the target of TNF-α adipocyte signaling. This displays the critical role TNF activation of NF-kB signaling can have on adipose homeostasis in a variety of contexts. As Tregs are master regulators of inflammation, they have also been observed to downregulate ILC2 activation in cases of allergic-type 2 inflammation in the lungs (100). However, as the inflammation is exacerbated in cases of allergy, Tregs have been shown to have a tissue-specific phenotype in the adipose (101). Due to a tissue-specific Treg phenotype in adipose, likely, ILC2s and Tregs work together to downregulate adipose tissue inflammation. Tissue-specific phenotypes of ILC2s and Tregs play a role in the maintenance of VAT homeostasis and ultimately determine glucose tolerance. In aging individuals, the number of adipose Tregs sharply increases and contributes to insulin resistance (102). The number of adipose ILC2s decrease and ILC2s become intrinsically defective due to aging; this directly contributes to thermogenic failure and insulin resistance in older individuals (103). Immunological mechanisms are likely a root cause of age-induced insulin resistance and T2DM, however, the interaction between Tregs and ILC2s in this development has not been fully investigated.

## Interactions Between ILC2s and Non-Immune cells in Prevention of T2DM

Adipose resident stromal cells are often multipotent present in the perivascular region of white adipose tissues (104, 105), and their communication with ILC2 is essential for the maintenance of metabolic homeostasis in the VAT. However, understanding the complex puzzle that is the cytokine milieu and its direct effects on the regulation and activation of ILC2s is challenging. Once trafficked to the adipose, IL-33 produced by adipose resident stromal and endothelial cells maintain ILC2 populations (98, 106). ILC2s explicitly function as a resident population therefore the maintenance and initial trafficking of them to adipose tissue are critical for their effects on the whole tissue. Visceral WAT adipose stem, progenitor, and mesenchyme-derived stromal cells produce IL-33 responsible for ILC2 activity and eosinophil populations (107–109). IL-33 licenses adipose tissue progenitor proliferation leading to the healthy expansion of adipose and homeostasis during a HFD. As tissue expands, healthy expansion of ILC2s *via* IL-33 producing stomal and stem cells is needed for proper homeostasis (107). Adipose multipotent stromal cells (MSCs) also respond directly to ILC2 derived IL-4 and IL-5 which upregulates their production of eotaxin and eosinophil recruitment to the adipose tissue (105). Therefore, ILC2s are able to regulate the accumulation of eosinophils to the adipose tissue through methods beyond IL-5 production; these cells enhance beige fat accumulation (47). Stromal cells are stimulated by cytokines IL-1β, TNF-α, and IL-17 (106, 110) which are interestingly found in higher concentrations in obese patients (111). Obese mouse models with long-term HFD have also noted an increase in IL-33 concentration (109). In the case of long-term obesity or HFD, the results of these studies seem to point towards induction of IL-33 to engage the early steps of type 2 immunity through activating ILC2s. However, as found by Oldenhove et al. (110) TNF-α and IL-33 together induce high PD-1 expression on ILC2s while increasing macrophage PD-L1 expression responsible for their dampened function in adipose tissue. The role of TNF-α as a potent indirect down regulator of ILC2 function is present even in presence of IL-33. As the immunological landscape changes, IL-33 ad TNF-α concentrations also change and consequently ILC2 numbers. Adipokines produced by resident adipocytes also interact with ILC2s. Leptin is a potent activator of lung ILC2s and induces type 2 cytokine production (112), however little is known about its role on adipose ILC2s. Adipocytes likely directly communicate with ILC2s through leptin production as they are the natural producers of this important adipokine. Understanding how different cytokines and age can regulate metabolic outcomes in adipose tissue is essential. The inability of cells to respond correctly to healthy signals may affect their ability to reverse harmful inflammation.

### ILC2s and the Nervous System in Adipose Tissue Homeostasis

The interaction between the nervous system and ILC2s in the adipose tissue is going to be a new research area. Recent reviews have discussed the role of ILC2s in communication with the nervous system and their impact on the regulation of pulmonary disease (113). ILC2s play a similar role in adipose tissue. The sympathetic nervous system releases catecholamines in response to stimuli, such as cold temperature, leading to the direct browning of adipocytes through thermogenic gene expression. Sympathetic nerves and innervation of the WAT are required for browning of white fat through regulation of ucp1 production (114, 115). Regional variation in PRDM16 expression between adipose tissue also correlates with sympathetic neurite density (116). It has been illustrated that brown ATMs control tissue innervation and energy expenditure (117). ILC2s have been shown to co-localize with nerves expressing the neuropeptide neuromedin U (NMU) and secrete type 2 cytokines (118–120). Intriguingly, 97% of resident immune cells expressing NMU receptor 1 (NMUR1) were ILC2s, showing that this receptor is selectively expressed (120). Further studies showed that NMU increases mRNA expression of *UCP1*, and rats with a genetic knockout of NMU showed less calorie expenditure (121). Recently, it has been shown that NMU promotes anti-inflammatory ILC2s and Tregs to protect against arthritis (122). Other neuropeptides like vasointestinal peptide (VIP), which increases in response to caloric intake, induces IL-5 and IL-13 production by ILC2s (123). The sympathetic nervous system directly stimulates IL-33 production and adipose ILC2s in response to cold exposure. When sympathetic denervation is induced, ILC2 and eosinophil accumulation is abrogated (124). The interface between ILC2s and the nervous system in adipose tissue is a critical battleground for T2DM pathogenesis.

In cases of T2DM, the chronic release of inflammatory cytokines such as TNF-α and IL-1β contributes significantly to peripheral neuropathy (125). In the same way that this causes pain for patients, neuropathy in the adipose tissue likely contributes to a lack of thermogenesis due to sympathetic nerve damage and lack of catecholamine production. ILC2s counter the production of type 1 cytokines through the promotion of M2 macrophages (18, 19, 21), helpful in maintaining adipose homeostasis. Interestingly, previous dogmas held that catecholamine production was the main inducer of thermogenic browning of adipose *via* ILC2s, however, this has been challenged (83). In confirmation of both this dogma and these findings, M2 macrophages indirectly, through the protection of sympathetic nerves, and directly, due to low catecholamine production, participate in thermogenesis in WAT *via* ILC2s. In addition, sympathetic neuron-associated macrophages have been found to import and metabolize NE contributing to obesity and adipose dysfunction (126). These cells are recruited and activated in obesity and have increased expression of TNF-α and IL-1. In another study, certain ATMs were also found to closely associate with tyrosine hydroxylase positive nerves, and higher age mice ATMs had high expression of catecholamine degrading enzymes (127). The effect of ILC2s on these inflammatory macrophages associated with nerves in adipose tissue has yet to be investigated. In the protection of nerves, M2 macrophages have been implicated as targets for the treatment of peripheral nerve injury through their ability to accelerate tissue repair (128, 129).

**Figure 1:**
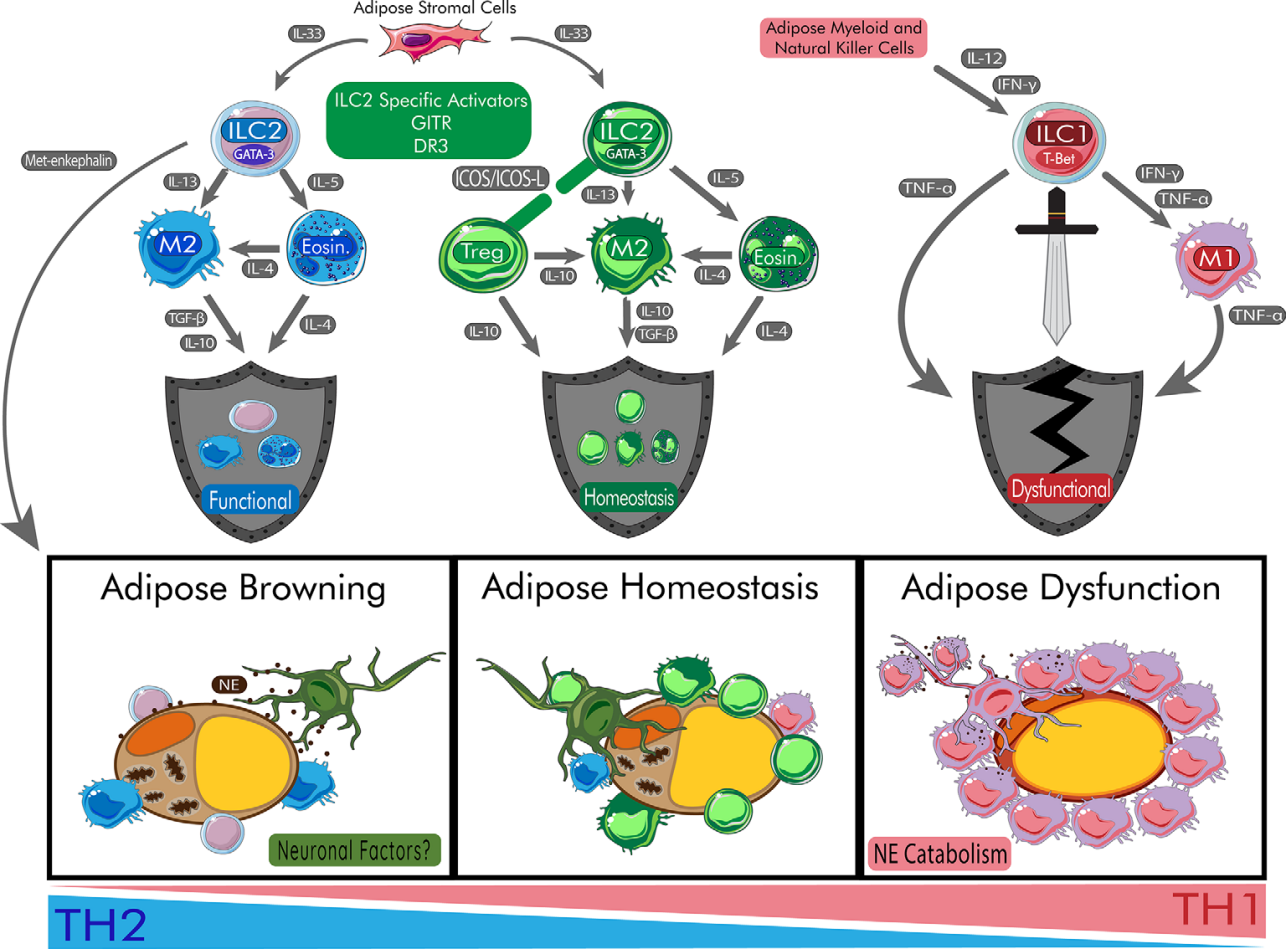
Role of ILC2s in Protecting Adipose Homeostasis and Promoting Beige Adipocytes. ILC2s begin a cascade of anti-inflammatory effects on the adipose tissue which allow for the browning of adipose tissue. During browning, adipocytes increase mitochondrial output and browning genes. Peripheral nerves are protected by ILC2s and communicate through neuronal factors such release of neuropeptides. In homeostasis, the balance of inflammation is maintained *via* healthy response to peripheral signals. During metabolic disturbances, the cytokines IL-12 and IFN-γ polarize ILC progenitors to become ILC1s responsible for damage of adipose tissue and systemic dysfunction. During this process, M1 macrophages are recruited to the adipose tissue and catabolize catecholamines like NE leading to insulin resistance. Chronic release of TNF-α also contribute to poor responses to insulin and T2DM. DR3, death receptor 3; Eosin, eosinophil; GITR: Glucocorticoid-Induced TNFR-Related protein; ICOS, inducible T-cell co-stimulator; IFN-γ, interferon gamma; IL, interleukin; ILC1, type 1 innate lymphoid cell; ILC2, type 2 innate lymphoid cell; M1, type 1 macrophage; M2, type 2 macrophage; NE, norepinephrine; T-bet, t-box transcription factor TBX21; TGF-β, transforming growth factor beta; TNF-α, tumor necrosis factor alpha; Treg, regulatory T cell.

The release of high levels of type 1 cytokines such as TNF-α and IFN-γ leads to neurodegeneration (130). In cases of T2DM, this may lead to suppression of thermogenesis in the adipose leading to systemic metabolic imbalance. ILC2 activation is severely diminished when sympathetic nerves are damaged (124). ILC2s are recruited to sympathetic nerves expressing NMU for the activation of immune responses (120). There, ILC2s would generate a protective type 2 and anti-inflammatory microenvironment around sympathetic nerves, which in the adipose tissue may protecting the production of catecholamines for thermogenic adipocyte differentiation. ILC2s co-localize and respond to androgenic neurons. These cells downregulate ILC2 activity through secretion of epinephrine through ILC2 expression of β_2_-adrenoreceptors (131). Exploration between the crosstalk and positioning of ILC2s in the adipose at nervous system interfaces requires more investigation. ILC2s do seem to be associated in some respect with neurons, seeking to communicate and react to CNS signaling in response to changing stimuli. As other reviews indicate (132), targeting of the neuron innate immune interactions offers many new therapeutic approaches for treatments of adipose tissue dysfunction including type 2 diabetes. ILC2s can mount a defense and protect neurons from damage caused by type 1 inflammatory responses present in obesity. With restored nerve responses, the adipose tissue can increase browning associated with better metabolic outcomes (**Figure 1**).

## Regulators of Adipose ILC2s

ILC2s maintain a high degree of regulatory control over immune cell types through the secretion of type two cytokines (18), but also interact with others cells through cell-to-cell receptor interactions. Unlike in mice, ILC2s in humans play specific roles in each localized tissue and likely display different effector functions due to site-specific transcriptional signatures (133). Activation of ILC2s in the fat would result in a therapeutic effect rather than in tissues such as the lung where they play a large role in the pathogenesis of asthma (10). Activation of ILC2s in WAT and VAT may provide a key part of T2DM treatments as their polarization of other innate populations may help ameliorate symptoms. Potent activators of adipose ILC2s such as tumor necrosis factor (TNF) superfamily receptors like GITR (20)and TNFRSF25 (DR3) (28, 134, 135) may be possible therapeutic targets for human T2DM treatment as their agonists promote adipose homeostasis and glucose tolerance in mouse models (20, 28). The involvement of receptors from other families such as ICOS (136, 137) and PD-1 (110, 138, 139) also provides some targets for sustained activation of ILC2s and promotion of Tregs and homeostasis (98). Although currently poorly understood in the VAT (10), the contributions of adhesion molecules, such as intercellular cell adhesion molecule-1 (ICAM-1) (140), and other cell-to-cell interactions are also crucial for the maintenance and activation of ILC2s (105). Maintaining crosstalk between stromal cells and ILC2s is essential for the browning of adipose tissue and the prevention of metabolic remodeling (141). Expansion of ILC2s in the VAT would be a therapeutic avenue as ILC2s decrease in number in cases of human obesity (142). Although ILC2s are the villain in the context of allergic diseases such as asthma, activation of these cells in adipose tissue plays a much different and helpful role through the prevention and attenuation of metabolic disturbance. ILC2s also maintain an elevated level of regulatory control over anti-inflammatory Treg populations (109). Their activation of Tregs is carried out through the expression of ICOS-L (98) and OX40/OX40-L (143) interactions. Current opinions hold that both ILC2s and Tregs are required for the maintenance of tissue homeostasis in the VAT (18, 101). Treatments that seek to target the cell-to-cell interactions between ILC2s and resident cells in the VAT would result in metabolic shifts able to alleviate insulin resistance.

### Positive Regulators of Adipose ILC2s

Adequate cell-to-cell interactions involving ILC2 activation are essential for their survival and sustained secretion of type 2 cytokines. Costimulatory molecules such as ICOS (136, 137) and GITR (20) are expressed on adipose ILC2s and healthy interaction with resident cells expressing ICOS-L and GITR-L respectively are essential for proper homeostasis. Studies have shown stimulation of GITR on ILC2s has been shown to directly protect against metabolic disturbances, such as type 2 diabetes, in obese mouse models through secretion of type 2 cytokines (20). Adipocytes were also observed to have decreased size and increased *UCP1* expression associated with higher energy expenditure and restored insulin sensitivity (20). Stromal cell expression of GITR-L (144) may provide stimulation for activated GITR^+^ lymphoid cells like ILC2s, but further specific exploration is needed. DR3 stimulation of ILC2s by cells expressing the ligand TL1A leads to their expansion, survival, and enhanced effector function (28, 134, 135). Unlike GITR (20), which is only expressed when activated, DR3 is constitutively expressed in naïve ILC2s making it a promising candidate for ILC2 activation and expansion in the adipose (28, 134, 135). DR3 stimulation also provides a more significant activation of human ILC2s in comparison to GITR (28). Recently, it was found that human cells from stromal cell vascular fractions in adipose tissue induced TL1A expression in response to TNF-related apoptosis-inducing ligand (TRAIL) significantly more in comparison to other cell types (145). TRAIL is commonly associated with a variety of obesity-related diseases and plays a role in human adipocyte inflammation (146). How DR3 and TL1A interact with ILC2s in human adipose requires further investigation.

Activated ILC2s express GITR which then seeks to sustain their activation. Treatments of activated ILC2s with DTA-1, a monoclonal agonistic antibody targeting GITR, were able to prevent metabolic disturbances induced by HFD, improve glucose homeostasis, and reverse established insulin resistance in mouse models (20). Increases in lean mass percentage accompanied with decreases in VAT mass were independent of caloric expenditure through caloric intake or physical activity. Through utilizing metabolic cages, it was shown that GITR activation also increased oxygen consumption and energy expenditure. Increased expression of *UCP1* and other associated calorie-burning genes were seen to be the cause of increased calorie expenditure and weight loss in the mice. DTA-1 treatment associated with higher levels of VAT M2 macrophages important for preventing metabolic disturbances (18, 21). The strongest evidence of ILC2 mediated protection against metabolic dysfunction was found through GITR^-/-^ mice with adoptively transferred WT ILC2s; DTA-1 treatment showed improvements in glucose tolerance solely mediated by activation of ILC2s through GITR engagement (20). Achieving specific and sustained activation of ILC2s would allow for a shift in glycolytically active tissues to a more stable and anti-inflammatory state able to prevent metabolic disturbances such as T2DM.

A more promising activator of ILC2s is DR3. Recently, a DR3 monoclonal antibody agonist, 4C12, was used in obese mouse models as a treatment to prevent metabolic disturbance and T2DM. The engagement of this receptor has shown effects in both naïve and activated ILC2s (28). Much like what was found with GITR (20), another TNF family receptor, DR3 engagement ameliorated glucose intolerance, protected the onset of insulin resistance, and reverse already established insulin resistance, but ILC2s displayed more significant effector function (28). Higher expression of adipose browning associated genes such a cell death activator CIDE-A (*CIDEA*), *PRDM16*, *PGC-1α*, cytochrome oxidase subunit VIIa polypeptide (*Cox7a*), and deiodinase 2 (*Dio2*) was detected in VAT lysates of DR3 agonist treated mice. The protective effect of DR3 was dependent on ILC2 secretion of IL-5 and IL-13; therefore, the observed secretion of IL-5 and IL-13 in human ILC2s with DR3 engagement in the study shows that DR3 may be a therapeutic target in human T2DM.

### Adhesion Molecules Regulate Trafficking and Maintenance of Adipose ILC2s

Adhesion molecules are some of the most important signals that regulate ILC2 function in adipose tissue. IL-33 induces the expression of ICAM-1 expression on human ILC2s and when stimulated leads to their activation. In mouse models, ICAM-1 deficiency led to attenuated ILC2 survival and development within inflamed lung tissues (147). Lymphocyte function-associated antigen 1 (LFA-1), the receptor for ICAM-1, has also been shown to be involved in the trafficking and survival of ILC2s. LFA-1-deficient mice were shown to have significantly less traffic to inflamed lung tissue; it also showed consistent expression across murine naïve and activated ILC2s unlike ICAM-1 which expression changed on activation (140). Likewise, *in vivo* blocking of beta-2 integrins, like LFA-1, led to significantly reduced ILC2s in blood and lung tissues (148). Determining what specific integrins regulate ILC2 accumulation in other tissues is critical. Deficiencies in adhesion molecules and their ligands consequently may be involved in the trafficking of maintenance of ILC2 populations in adipose tissue. Soluble forms of ICAM-1 have also been observed to increase in obese mouse models fed HFD leading to increased macrophage recruitment due to expanding fat mass (149). However, in other HFD fed mice, leukocyte migration to the adipose was not affected by ICAM-1 deficiency (150). Adipose tissue-resident multipotent stromal cells (MSCs), while acting as a reservoir of IL-33 for ILC2s, also stimulate them through the expression of ICAM-1 inducing proliferation and activation in LFA-1 expressing ILC2s (105). ICAM-1 depletion in the adipose MSCs leads to impaired ILC2 proliferation and effector function in the WAT; knockdown of LFA-1 on ILC2s resulted in the same findings. The ICAM-1/LFA-1 axis on ILC2s has a clear role in the regulation of adipose tissue homeostasis.

### Negative Regulators of Adipose ILC2s

The role of immune checkpoints in the adipose tissue regarding ILC2s is becoming increasingly clear. PD-1 is directly involved in the function of ILC2s in a variety of contexts (110, 138, 139). In the adipose tissue, PD-1 is upregulated in IL-33-activated ILC2s in response to TNF-α, present in high concentrations due to obesity (110). In addition, TNF-α secretion by resident ATMs recruits and activates PD-L1^hi^ M1 macrophages further dampening ILC2 responses in the adipose tissue (110). In agreement with previous findings (18, 19), HFD and obesity decreased numbers of IL-13+ ILC2s, M2-like ATMs, and eosinophils (110). The number of IL-5/IL-13+ ILC2s did were unaffected by HFD in TNF-α KO mice, with the same trend being observed with M2-like ATMs (110). TNF-α injection further impaired ILC2 function and upregulation of PD-1 expression *via* IL-33, while depletion of inflammatory M1-like macrophages rescued ILC2s. PD-1 blockade also partially restored the ILC2/AAM/Eosinophil axis, enhanced *UCP1* expression, and ameliorated glucose intolerance during a HFD (110). This study highlights that TNF signaling regulates two crucial steps in adipose tissue homeostasis *via* ILC2s. The first is the induction of PD-1 expression on ILC2s indirectly *via* IL-33, and the second is the activation of the differentiation and recruitment of M1-type macrophages from the periphery which express high PD-L1, unlike M2-like ATMs. Restoration and protection of ILC2 responses in cases of HFD and obesity are regulated by the crucial PD-1 axis.

Adiponectin, an important adipokine, was shown to restrain adipose ILC2 activation *via* AMPK (151). Interestingly, adiponectin has a controversial role in energy expenditure and diabetes with conflicting studies over its role as a pro- or anti-thermogenic signal. In this study, adiponectin knockout suggested an inhibitory effect on thermogenesis and a pro-adipogenic role (151). It was found adipose thermogenesis and energy expenditure were dependent on ILC2s as adiponectin levels decreased with cold temperatures (151). AMPK is activated by IL-33 in adipose ILC2s and acts as a feedback inhibition mechanism for the induction of NF-κB and therefore GATA-3 and ILC2s (151). In addition to acting on resident adipocytes and stromal cells, adiponectin was an immunological mediator stopping ILC2 mediated thermogenesis.

Cell-to-cell interactions mediated by other adhesion molecules are key in the production of IL-33 and can have an indirect effect on ILC2 functions in adipose tissues. Mesenchymal cadherin-11 directly downregulated stromal cell production of IL-33; cadherin-11-deficient mice displayed increases in IL-13 and M2 macrophage differentiation and expansion in adipose (152). It is possible that in some cases of obesity-induced inflammation there may be cadherin-11 overexpression leading to less IL-33 in adipose tissues. Lack of sustained activation of ILC2s following may lead to decreased number and effector functions. E-cadherin, the ligand for killer cell lectin-like receptor subfamily G member 1 (KLRG_1_) expressed on ILC2s, has also been shown to downregulate ILC2 cytokine secretion and activation, though it was found in the context of atopic dermatitis (153). However, the expression and *in vivo* relevance of E-cadherin has yet to be examined in adipose tissues of patients with T2DM.

One possible contribution to insulin resistance is genetic differences in diabetic patients that lead to dysfunctional ILC2s that are unable to maintain healthy adipose homeostasis. One of the largest genetic components of this is the contribution to autophagy. It has been demonstrated that autophagy-deficient ILC2s lead to decreased cytokine secretion and increased apoptosis (154). These would both contribute to a lessened type 2 response leading to a predominantly type 1 environment in adipose tissues that would disrupt its balanced homeostasis. Intriguingly, autophagy deficiency has also been shown to disrupt innate lymphoid cell development and trafficking. Lymphocyte survival following homeostatic proliferation required autophagy (155). ILC2 precursors, deficient in autophagy and being exposed to a highly inflammatory cytokine milieu, may be unable to reach their target tissues due to induction of apoptosis for failing to meet their energetic needs. Obese patients have been shown to have altered autophagy which may contribute in some patients to diabetes. In these cases, it seems that autophagy can be either enhanced or attenuated in different patients contributing to their obesity; therefore, the heterogeneity of the human population may contribute to some obese patients developing T2DM or not (156). Other polymorphisms involved in ILC2 function may also affect their ability to fight T2DM. As observed with *ST2* deficiency in mouse models (157), brown adipocytes displaying polymorphisms in their *ST2* gene may be a cause of inadequate ucp1 protein product. This could have been mediated by the *ST2* deficiency in ILC2s leading to their inability to induce *UCP1* expression like observed in Galle-Trager et al. (20). Although most studies of ILC2s focus on the effects of cell autophagy on lung inflammation (158), there are still questions as to how autophagy in adipose tissue ILC2s can contribute to T2DM pathogenesis. Likely, genetic differences in the human population, especially in all-encompassing genes such as autophagy-related genes, show connections to obesity and ultimately may contribute to the pathogenesis of T2DM.

### Virus-Induced Interferon and Dampened ILC2 Function

Interferon signals activate STAT1 for the transcription of genes for a more specific response to viral infection. A patient is nearly four times more likely to develop T2DM if they are infected with hepatitis C virus (HCV) (159). Successful treatments of HCV in T2DM patients displayed improvements in glycemic control and resulted in less insulin use by patients (160). Innate immune cells such as dendritic cells are responsible for the production of IL-12 in response to HCV infection through TLR-3 signaling leading to NK cell production of IFN-α (161). High IL-12 results in ILC1-like ILC2s with the expression of T-bet (162). These ILC1-like ILC2s produce IFN-γ which further diminishes committed-ILC2 effector function (75, 76, 98). Meta-analysis has shown that HCV infection is associated with an increased risk of T2DM independent from associate liver disease (163). The true cause of this association has yet to be observed, however immunological mediators of the pathogenesis of HCV are likely involved such as ILC2s. In addition, any production of IFN, type 1 or IFN-γ, due to HIV infection may be a causative agent in T2DM comorbidity; patients infected with HIV are also up to four times more likely to have T2DM (164). Plasmacytoid dendritic cells (pDCs) have been shown to produce copious amounts of IFN-α in response to HIV (165). The link between viral infection and the diagnosis of T2DM is still being investigated.

Interferon originating from peripheral locations can lead to restricted Th2 and Th17 responses, including that from ILC2s (166). STAT1 deficiency leads to a lack of proper response to pathogens, but overactivation of the transcription factor can lead to autoimmunity and sustained inflammation due to enhancement and positive feedback in IFN-α/β signaling (167). Genetic gain of function in STAT1 has not been investigated in metabolic diseases such as T2DM, although interferon would lead to decreases in ILC2s (75, 76, 98). Classically, the survival of ILC2s relies on stimulation by IL-2 and IL-7 as well as their functional receptors. Genetic defects in these signaling pathways leading to STAT activation may contribute to ILC2s’ inability to maintain adipose homeostasis and insulin sensitivity. STAT6 activation through IL-4 receptors (IL-4R) plays a key role in homeostasis and cytokine production in ILC2s. Mice deficient in STAT6 have been shown to have impaired numbers of IL-13 producing ILC2s (168), a phenotype usually displayed in IL-4R deficient mice. More genetic studies in humans are needed to quantify the effects of polymorphisms of specific transcription factors for their implications into ILC2 mediated adipose inflammation and T2DM.

## Plasticity and Human ILC2s

The plasticity of ILC2s and other innate lymphoid cell populations is an important issue to address when discussing their roles in T2DM. While not fully investigated plasticity of adipose ILCs likely plays a key role in protection against disease or pathogenesis. ILC1s are associated with adipose inflammation and have been suggested to be responsible for most of the IFN-γ production in the case of HFD (169, 170). ILC2s are able to adapt to an ILC1-like phenotype to further enhance inflammation. Similarly, ILC2s adopting an ILC3-like phenotype may also be implicated in obesity and metabolic homeostasis through secretion of IL-17 and IL-22 (171). ILC3-derived IL-17 is associated with obesity-induced AHR (172) and there may be pathological accumulation in other tissues as well. In cases of T2DM, IL-22 alleviates metabolic dysfunction through the reduction in islet β-cell inflammation and promotion of liver homeostasis (173, 174). The role of ILC3s in adipose homeostasis has yet to be fully understood, though recent studies utilizing single-cell RNA sequencing have identified that ILC3s are mediators of human adipose tissue inflammation *via* the expression of inflammatory mediators (175). The ideal treatment would be the suppression of ILC1 and ILC3 phenotypes in adipose tissues. Some ILC3s, while mainly defined by the expression of RORγt, also can express T-bet which is the transcription factor associated with ILC1s (38). This leads to their production of IFN-γ and the promotion of type 1 inflammation. The transcription factor c-Maf regulates T-bet expression and therefore is the determinant of the ILC1 (T-bet) or ILC3 (RORγt) phenotype (176). It is currently unknown the effect that IFN-γ+ ILC3s have in diabetically active tissues. The induction of ex-ILC2s is dependent on the induction of T-bet which is connected to cytokines IL-1β, IL-12, and IL-18 (177). Likewise, notch signaling due to fungal infection in mice induces the upregulation of RORγt and IL-17A in ILC2s (178). Humans display ILC2 to ILC3-like conversion when stimulated with cytokines IL-1β, IL-23, and TGF-β (179). Further study is necessary to fully understand how adipose ILC2 and other innate lymphoid cell populations can change phenotype due to different inflammatory environments such as obesity. Making these studies more challenging is that ILC3s are not present in lean or obese mouse WAT (169).

ILC2s in human disease have a very distinct role to play in the prevention of chronic adipocyte stress and inflammation responsible for the pathogenesis of T2DM. Anti-TNF treatments have shown limited success in restoring insulin sensitivity in patients with T2DM (180) which requires that other pathways need to be explored in treatments. Recent data has shown that, unlike ILC1s or ILC3s, ILC2s seem to have higher plasticity from ILC progenitor cells; the fates of ILC1s and ILC3s in humans seem to display a clear developmental trajectory (175). However, while the trajectory is more defined, once human ILCs arrive at their tissue they still maintain the capacity to adopt traits from other ILCs. Human ILC3s from mucosal tissues have been able to produce IFN-γ, like ILC1s, *in vivo via* transcription factors T-bet and Aiolos (181). Likewise, human ILC1s can adopt ILC3-like capacities *in vitro* in the presence of IL-23 (182). Human ILC2s also can be skewed towards an ILC3, IL-17 producing, phenotype by IL-1β, IL-23, and TGF-β stimulation (179). IL-1β seems to be a key regulator in unlocking ILC3-like functions in human ILC2s as just IL-23 alone is not able to induce IL-22 production by ILC2s (183). When human ILC2s adopt ILC1 capacities they only need IL-12 (183). Understanding human ILC progenitors and how they develop into the different phenotypes still requires more research as the plasticity of these populations in tissues remains relatively high compared to other cells. Maintaining the ILC2 phenotype *via* metabolic or pharmacological intervention would be essential for protection against T2DM pathogenesis.

## Metabolomics of ILC2s

Diet is a major component of obesity and T2DM as concentrations of metabolites such as glucose and different fatty acids fluctuate. ILC2s have been shown to depend on fatty acid metabolism for their effector function in multiple contexts (154, 184–187). ILCs acquire long-chain fatty acids from the environment and adipose resident ILCs have the highest uptake across multiple peripheral sites (186). While ILC2s at a steady state are not uniquely dependent on fatty acid oxidation, metabolic requirements change upon activation as the need for energy increases. In response to helminth infection, ILC2s protective function is directly dependent on fatty acid availability and their ability to process them for energy (186). ILC2s were further found not to be reliant on glycolysis but were impaired by lack of fatty acid oxidation through the utilization of etomoxir, a fatty acid oxidation inhibitor, and orlistat, a lipase inhibitor (186). As ILC2s uptake external lipids, regulated by the expression of PPARγ and diacylglycerol o-acyltransferase 1 (DGAT1), the molecules are stored in lipid droplets and converted into phospholipids which aid in proliferation (187). Due to excess fatty acids accumulated in these active ILC2s, DGAT1 expression is necessary to avoid lipotoxicity through the formation of lipid droplets.

**Figure 2:**
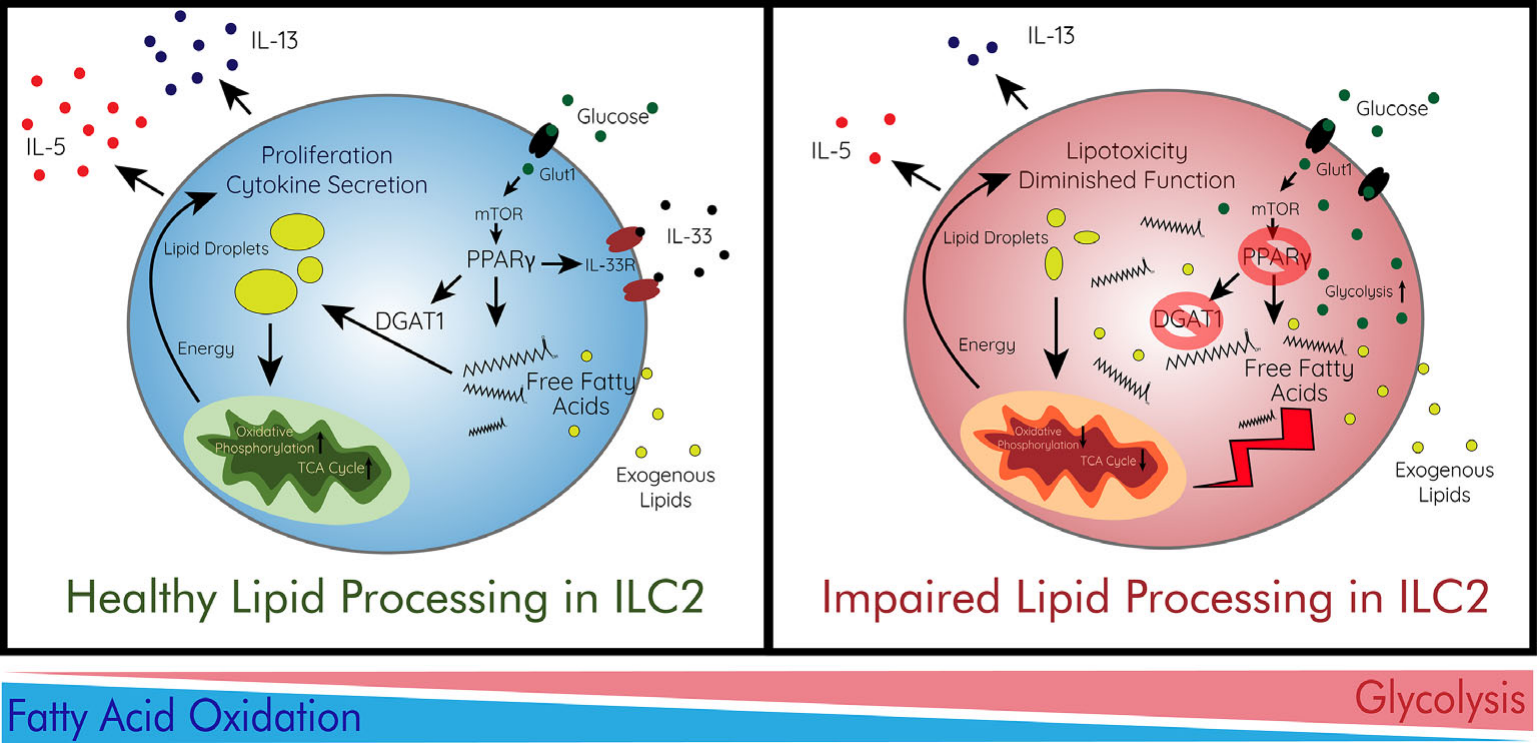
Immunometabolism and Lipid Processing in ILC2s. ILC2s preferentially utilize fatty acid oxidation for their proliferation and cytokine secretion. In order to survive in the adipose tissue, ILC2s avoid lipotoxicity *via* storage of free fatty acids in lipid droplets. This process is regulated by the energy sensor mTOR which controls PPARγ and DGAT1 which ILC2s use to store fatty acids in droplets. mTOR is largely regulated by the abundance of glucose imported by Glut1. ILC2s then increase oxidative phosphorylation for their production of energy to enhance their function. When lipid processing is impaired, free fatty acids accumulate heavily in adipose ILC2s leading to lipotoxicity and diminished function. This can be caused by deficiencies in PPARγ or DGAT1. In addition, ILC2s intake more glucose and increase glycolysis as they are unable to utilize fatty acids effectively. This immunometabolic switch leads to decreased effector function and survival of ILC2s. Glut1, glucose transporter 1; IL, interleukin; IL-33R, IL1rl1; ILC2, type 2 innate lymphoid cell; mTOR, mammalian target of rapamycin; TCA cycle, citric acid cycle.

PPARγ is highly implicated in the production of IL-5 and IL-13 by ILC2s and inhibition drastically reduces their effector function (187, 188). Dominant-negative mutations in human PPARγ are associated with insulin resistance and T2DM (189). PPARγ additionally is required healthy function of adipose ILC2s as antagonist-treated and PPARγ-deficient mice displayed greatly diminished number, frequency, and effector functions (190). PPARγ also regulates the metabolism of ILC2s *via* the enhancement of CD36, a fatty acid importer, and the expression of IL-33 receptor (190, 191). PPARγ is a master regulator of fatty acid and glucose metabolism, and its implications for ILC2 metabolism and activation show how metabolism and cell activation states are deeply rooted in each other. PPARγ expression in ILC2s likely has some role to play in this development of insulin resistance and disease due to its regulatory role of metabolism and cell activation (**Figure 2**).

As previously discussed, PD-1 is important in the protective effect of ILC2s and M2 macrophages on metabolic outcomes. PD-1 has recently been shown to be important in the metabolic function of ILC2s as well. ILC2s with PD-1 knockout displayed increased expression of Glut-1, the main glucose transporter, and enhanced aerobic glycolysis instead of fatty acid oxidation which ILC2s preferentially utilize (139). PD-1 was also shown to regulate methionine and glutamine catabolism as well as regulate ILC2 proliferation *via* metabolic mechanisms (139). What other immune checkpoints and cell-to-cell interactions play a role in the metabolism of ILC2s has yet to be investigated. The immunometabolism of ILC2s is becoming increasingly important in the regulation of their functions and roles in disease treatments.

## Novel Targets for Amelioration of T2DM

There are a variety of current treatments that help to manage hyperglycemia in T2DM, however, none have sought to treat the disease in a cell-specific manner. Beyond diet and exercise (192), different drugs have been used to treat T2DM. Exogenous insulin has long been a diabetes treatment, in cases of high insulin resistance, this would become increasingly less effective. Thiazolidinedione class drugs seek to activate PPARγ in adipose to alter adipose metabolism and distribution of triglycerides (193). These drugs also reduce circulating TNF-α and IL-6 concentrations (194), while maintaining high concentrations of adiponectin responsible for insulin sensitization (195). Metformin has long been used to suppress hepatic glucose production, but its full mechanism is still being fully elucidated (196). Alpha-glucosidase inhibitors seek to delay glucose absorption from the upper gastrointestinal tract leading to glycemic control (197). Sulfonylurea derivatives close pancreatic cell potassium channels enhancing insulin secretion (194). While these drugs can manage T2DM, they do not seek to treat the issue at its source. Therefore, there is still a need for more novel treatments seeking to provide long-term relief for T2DM patients.

Specific regulators of immunity are needed for the amelioration of T2DM and understanding how activation of ILC2s can contribute to those treatments are essential for maintaining metabolic homeostasis. There are a variety of treatments that seek to target adipose tissue in the treatment of obesity-associated diabetes and T2DM as reviewed by Kusminski et al. (56). However, other immunological mediators of adipose inflammation have yet to go through proper research and development for humans. Novel regulators of ILC2 function such as GITR (20) and DR3 (28) provide clear avenues for the amelioration of insulin resistance and establishing glucose tolerance in obese individuals. *In vivo* drug treatment has been shown to regulate human adipose ILC2 populations (198). Overall, more immunotherapeutic strategies need to be adapted to complex diseases such as T2DM as the presence of immune dysregulation is a key driver in their development and pathogenesis.

GITR has come into interest for immunotherapies for a variety of different diseases including diabetes (199). In mouse models, it seems that treatments with GITR agonists DTA-1 (20) and G3c monoclonal antibody (200) are able to help treat type 2 and type 1 diabetes respectively. G3c monoclonal antibody was shown to activated islet-specific Treg cells for downregulation of inflammation in NOD mice which leads to islet β-cell death (200). Though it is a promising target for these diseases, the different molecular structures and assembly of human and mouse GITR/GITRL may make translating treatments challenging. In clinical trials utilizing GITR agonistic antibody TRX518 for cancer treatments, only 37% of patients experienced treatment-related adverse events, most often fatigue (11.6%), with no dose-limiting toxicities or serious adverse events (201). This trial shows that GITR modulation in human clinical trials is safe and may be able to be translated to diabetes treatments in the future. Adding complication, anti-murine GITR mAbs display high agonistic effects while several anti-human GITR mAbs display weak agonistic or even antagonistic effects (202). This is likely due to murine GITRL forming a dimer in solution, while human GITRL forms very weak tertiary structures needed for activation of the hGITR receptor. Overall, the development of more hGITRL antibodies are needed to imitate *in vivo* tertiary structures able to activate hGITR expressing cells.

Finding receptors responsible for the activation of ILC2s are essential in the control of homeostasis in cases of T2DM. Targeting both inducible (20) and constitutively expressed receptors such as DR3 (28) can provide activation needed for the amelioration of the disease. Constitutively expressed receptors provide a promising target in cases of T2DM due to most cases involving limited activation of ILC2s as type 1 immunity is predominant. Treatments involving other immunomodulatory agents such as prostaglandins may provide ways to regulate both trafficking and activation of ILC2s (94, 203). Constitutive CRTH2 expression on a variety of cells, not only ILC2s, provides different therapy options for a variety of diseases (203). In the context of T2DM and adipose homeostasis, the effects of CRTH2 involved treatments are unknown in the context of immunity, although agonists have been found to regulate lipid accumulation in adipocytes (204). Depletion of ILC2s through CRTH2 antagonists in cases of asthma have shown to be therapeutically viable in mouse models (94) and promising in human studies (205). When seeking the opposite pathological effect in cases of T2DM, in adipose tissues agonists of CRTH2 may enhance activation of ILC2s and general type 2 immunity. In general, targeting of constitutively expressed receptors provides a better option for treatments than their inducible counterparts as some phenotypes of human T2DM may not display activation necessary for the inducible expression of co-stimulatory molecules.

Reagents that seek to target pathways that indirectly tip the immunological balance away from type 2 immunity through downregulation of ILC2s may be a possible treatment for T2DM. As STAT1 mediated IFN-γ signaling can both affect adipocytes (206) and ILC2s alike (75, 76), drugs able to regulate STAT1 signaling in the adipose tissue could be a key factor in regulating adipose homeostasis. STAT1 inhibitors have been tested in a variety of contexts including atherosclerosis, encephalomyelitis, and other autoimmune diseases as a means to promote immunological balance through deregulating an overreaching type 1 response (207). Besides their effects directly on ILC2s and type 2 immunity, STAT1 and JAK inhibitors also have direct effects on adipocytes. JAK inhibition with pharmacological inhibitors acquired more brown-like metabolic profiles mediated by downregulation of IFN-α/β/γ signaling (208). Short-circuiting JAK/STAT1 signaling leads to a lack of positive feedback needed for adipose whitening or anti-browning. The development of hydrophobic pharmacological inhibitors of STAT1 may be a way to increase tissue specificity. These drugs would have a synergistic effect through attenuation of IFN-α/β/γ signaling in adipocytes and ILC2s dampening WAT inflammation.

## New Perspectives of ILC2s in T2DM

### Trafficking, Retention, and Maintenance of ILC2 in Inflamed Adipose Tissue

Though adipose ILC2s are considered tissue-resident cells with low-level replenishment from the periphery (12), in cases of inflammation the trafficking of ILC2s changes (13). The ability of ILC2s to traffic to inflamed tissues, such as adipose tissues, is essential for the prevention of insulin resistance. Overall, more studies need to be done to fully understand the process of ILC2 trafficking to multiple peripheral tissues. Expression of CRTH2 and its ligand PGD_2_ regulates chemotaxis of ILC2s to the lungs in the context of allergic asthma (94, 95, 209). Stimulation of mineralocorticoid receptors, often associated with blood pressure dysregulation, has been shown to increase adipocyte PGD_2_ synthase (210), which could regulate ILC2 accumulation. However, the chemotaxis of ILC2s to diabetic active tissues still requires more research. Other receptors such as cysteinyl leukotriene receptor 1 (CysLT1R) on ILC2s and leukotriene D_4_ (LTD_4_) concentrations may also regulate chemotaxis to inflamed tissues (211). Leukotrienes (LT) like LTC_4_, LTD_4_, and LTE_4_ all were shown to induce migration, cytokine secretion, and reduce apoptosis in ILC2s; LTE_4_ was specifically shown to enhance effects of PGD_2_, IL-25, and IL-33 (212). How these chemotactic markers work in trafficking ILC2s to adipose is still up for debate.

One recent subject of interest has become mature ILC2 trafficking to and between diverse tissue sites. ILCs are largely found as tissue-resident cells (11), but through migrations, they can regulate systemic homeostasis. IL-25 has been shown to regulate the trafficking of KLRG1^High^ inflammatory ILC2s (iILC2) from the small intestine to the lungs through differential expression of sphingosine 1-phosphate (S1P) receptors (13). This traffic was found to be both to lymphatic and non-lymphatic organs, with a substantial number of ILC2s originating from the gut. Interestingly, the results indicated that intestinally derived iILC2s infiltrate the bone marrow where they can have effects on ILC2 progenitors (13). Although IL-25 has been shown to stimulate a type 2 inflammatory response associated with M2 macrophage polarization adipose tissue of mice (213), natural sources of IL-25 in the adipose remain elusive. However, injections of IL-25 in obese mouse models display enhance *UCP1* expression, M2 macrophage polarization, and overall improvements in glucose homeostasis (84), showing that activation of ILC2s in adipose may provide a route of treatment for T2DM. Disrupted trafficking of ILC2s between organs in cases of T2DM inflammation still needs to be further investigated.

**Figure 3:**
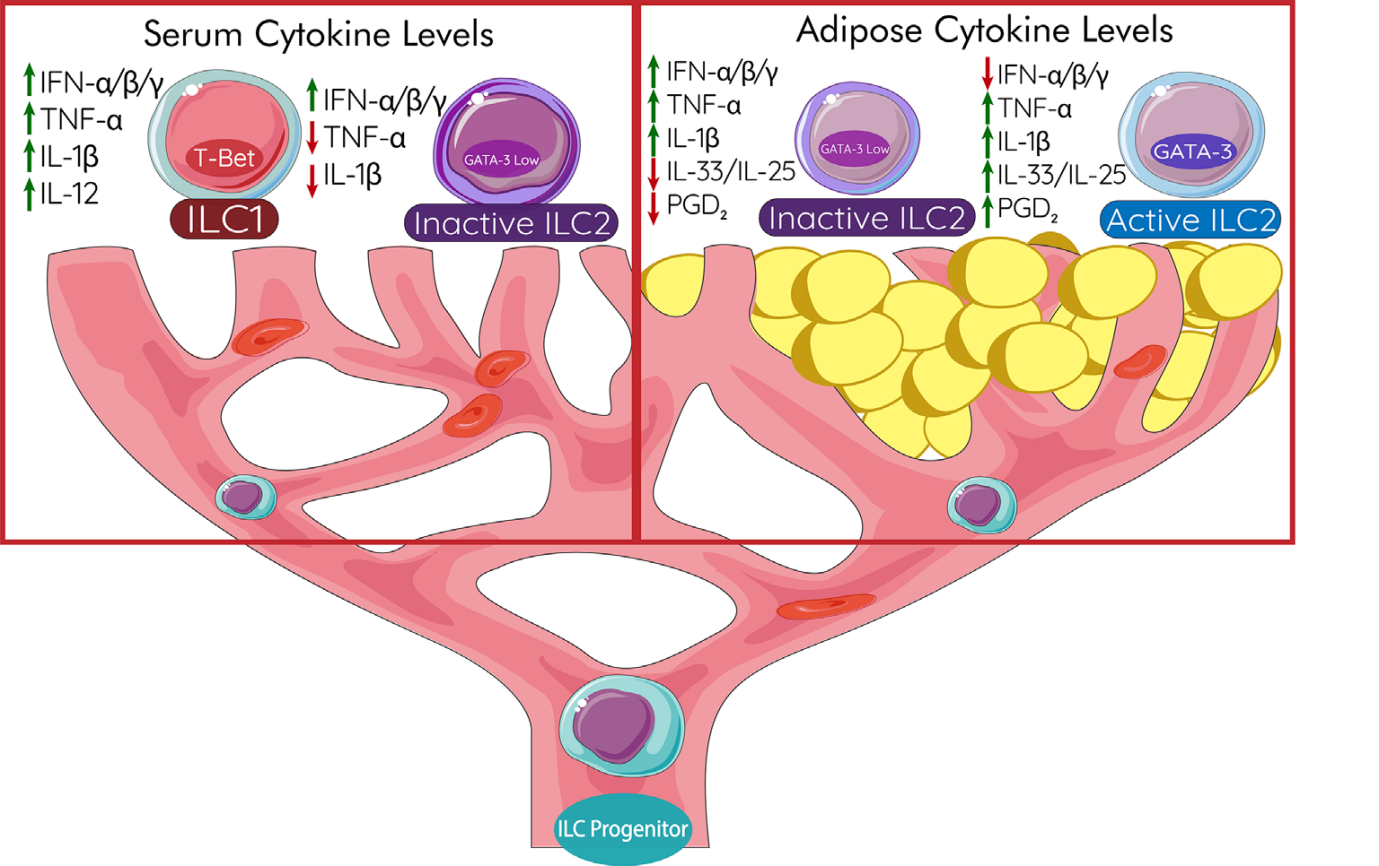
ILC2 Trafficking and Maintenance in Adipose Tissue. ILC progenitors are able to traffic from the bone marrow to their resident tissue to replenish and restore type 2 responses. In the blood, cytokine levels affect the outcome of these progenitors due to their natural plasticity. ILC progenitors exposed to high IFN, TNF-α, IL-1β, and IL-12 will develop into ILC1s. While ILC2s could still develop, high IFN-α/β/γ levels greatly decrease the effector function of ILC2s rendering them inactive. Once arrived in the adipose tissues, low IL-33 and PGD2 effects the number and function of ILC2s. High TNF-α, IL-1β, IL-33, and PGD_2_ generate active ILC2s able to protect adipose from dysfunction responsible for T2DM. IFN, interferon; IL, interleukin; ILC, innate lymphoid cell; ILC1, type 1 innate lymphoid cell; ILC2, type 2 innate lymphoid cell; PGD_2_, prostaglandin D2; T-bet, t-box transcription factor TBX21; TNF-α, tumor necrosis factor alpha; Treg, regulatory T cell.

Depending on the type of immune cell their destination and outcome due to the cytokine and chemokine milieu in the blood can be completely different. ILC2s exiting the bone marrow are met with a variety of cellular signals which dictate their outcomes (**Figure 3**). In cases of systemic inflammation, such as obesity-induced diabetes, pro-inflammatory cytokines can be present in the blood and concentrated in fat tissues as activated adipose tissue macrophages (ATMs) produce tumor necrosis factor-alpha (TNF-α) and interleukin-1β (IL-1β) (111). These cytokines lead to the activation of ILC2s for the production of cytokines IL-5 and IL-13 (162, 214), however, due to the plasticity of ILC2 populations, the strong presence of these cytokines can also lead to the conversion of these cells into ILC1 phenotypes (162) (ex-ILC2) to sustain the type 1 immune response. In cases of obesity, ILC2 progenitors exiting the bone marrow may be met with strong signals from type 1 cytokines leading to their differentiation into an ILC1-like phenotype. Interestingly, IL-12, a requirement for T-bet^+^ ILC2s (162), is elevated in obese patients in a variety of studies (215–217) showing that obese patients may show a suppression of the ILC2 phenotype. ILC1s are directly linked to the pathogenesis of T2DM through inducing adipose fibrosis and M1 macrophage activation (170). Adipose-resident ILC1s also promote insulin resistance in cases of obesity through the production of IFN-γ necessary for pro-inflammatory macrophage polarization (169). Production of TNF-α by ILC1s would lead to further exacerbation of insulin resistance which has a variety of downstream consequences for insulin signaling. These directly affect serine kinases (IKK, JNK, S6 Kinase) and mTOR which are involved in attenuating insulin signaling and healthy response to the hormone (86).

Interestingly, interferons like IFN-γ and interleukin 27 (IL-27), have been shown to directly restrict ILC2 activation, cytokine production, and increase apoptosis (75, 76, 98). Activated natural killer cells (NK cells) are often the main source of IFN-γ in the adipose tissue and its secretion leads to pro-inflammatory macrophage accumulation. NK cells are directly activated in cases of obesity through the upregulation of NK cell-activating receptors on adipocytes; more specifically through the expression of NKp46, known as natural cytotoxicity triggering receptor 1 (NCR1) in mice (218). They also directly contribute to insulin resistance in obesity (219). Circulating cells like pDCs produce a copious amount of IFN-α which has been shown to regulate ILC2 functions. IFN-α from pDCs has been shown to directly suppress cytokine production, proliferation, and increase apoptosis in ILC2s (220). Circulating cells such as pDCs therefore may play a role in ILC2 progenitor development through IFN-α signaling. Signal transducer and activator of transcription 1 (STAT1), a downstream transcription factor of IFN-γ signaling, plays a key role in the control development of adipocytes and the ability of interferon to induce insulin resistance (206). Therefore, interferon production is a key player in adipose inflammation and homeostasis, not only circulating ILC2 progenitors. High circulating interferon would polarize ILC progenitor towards an ILC1 phenotype. Halting other phenotypes of innate lymphoid progenitors and promoting ILC2s is essential in maintaining healthy adipose tissue homeostasis and preventing metabolic remodeling leading to insulin resistance.

Trafficked naïve ILC2s to the inflamed adipose are key in the regulation of the cytokine profile and outcome of the tissue. Maintenance of arrived ILC2s is dependent on pro-survival signals. One of the most potent activators of not only activated but naïve ILC2s is DR3 (28, 134, 135). In contrast to GITR (20), DR3 activates ILC2s through both canonical and non-canonical NF-κB pathways (28). It is therefore plausible that the observed increase in mouse VAT ILC2 activation, when stimulated with DR3 agonist, is due to this doubled activation of the NF-κB pathways. Treatments seeking to maintain ILC2s in the adipose through activation of DR3 may be a possible therapeutic route for T2DM treatment. Antigen-presenting cells in the VAT expressing TL1A (221, 222) also may be able to specifically stimulate newly trafficked ILC2s in the adipose tissues and therefore would be able to regulate their numbers. Although TL1A expression has been explored in a variety of different contexts (221), the role of TL1A in adipose has yet to be fully explored. In one study, TL1A-deficient HFD mice showed decreased adiposity, glucose tolerance, but also reduced expression of *UCP1* (223). It was also found that TL1A deficiency also reduced the abundance of ILC1s. Interestingly, these findings indicate that the effect of TL1A is dependent on the context. Obesity conditioned adipose may be receptive to DR3 agonists. However, in a predominant type 1 inflammatory environment, the presence of a DR3 agonist may be detrimental as it would stimulate more ILC1s than ILC2s. Therefore, the timing of these treatments and understanding the inflammatory phenotype of the patient is extremely important. The ability to shift the adipose microenvironment towards a more Th2 cytokine profile and predominant ILC2s would likely prove helpful if treatments utilizing DR3 agonists were to be considered. Early-stage treatment for T2DM would therefore require DR3 antagonization for increases in glucose tolerance and insulin sensitivity, followed by DR3 activation for the sustained maintenance of adipose trafficked ILC2s and increase in *UCP1* expression and adipose browning. Treatments seeking to traffic and maintain ILC2s in the adipose tissue will play a key role in future treatments of T2DM and conditions of adipose deregulation.

### ILC2s, Leaky Gut, Microbiota, and T2DM

Other genetic factors leading to the release of interferons can regulate populations of ILC2s through dampening their effector function. A recent hypothesis in the pathogenesis of T2DM is that a leaky gut leads to systemic inflammation through the release of interferons. In addition to the leaking of short-chain fatty acids and bile acids leading to insulin resistance, the bacteria and LPS leaking out of the gut leads to the release of a variety of inflammatory cytokines from immune cells (224, 225). Cytokines like IFN-α/β can leak into the bloodstream and spread peripherally negatively affect ILC2 number and function (75, 76, 98). The microbiota is being explored in a variety of contexts due to its role in the initiation of gut inflammation. ILC2s in the gut have many interactions with bacteria, however, their contribution to T2DM has yet to be explored fully. Commensal microbiota has been observed to impact epigenetic regulation and gene expression of ILCs (226). Consequently, there are likely genetic and external components of the gut microbiota that may affect the inflammatory outcomes of ILC2s and T2DM on a more systemic scale. There is a new and upcoming role of microbiota in the understanding of immunity and the development of seemingly unrelated diseases like T2DM.

### ILC2_10_s

Relatively new in the study of innate biology, ILC2_10_s, IL-10 secreting ILC2s, have been implicated in a variety of inflammatory contexts (227). ILC2s have been observed to secrete IL-10 in lungs (228–230) and gut tissues (231). It was recently discovered that ILC2_10_s express transcription factors cMaf and Blimp-1 for IL-10 secretion in the context of allergic lung inflammation (229). IL-10 production by ILC2s has also been inducible by NMU (231), emphasizing the importance of the sympathetic nervous system in ILC2 regulation. Although it is currently unknown, these regulatory ILC may play a role in the downregulation of inflammation in the adipose. As the relationship between type 1 and type 2 inflammation is dichotomous, secretion of IL-10 and Treg cells ultimately leads reduced overall immune activation and complete homeostasis. It is reasonable that ILC2_10_s may play a key role in more long-term outcomes of T2DM patients and downregulation of all types of inflammation in adipose tissue. Interestingly, they have also been shown to reduce *in vivo* eosinophil recruitment (228). Discovery of this very plausible population of ILC2_10_s in cases of extreme adipose inflammation is needed as the expansion of this population would likely provide a treatment option for T2DM.

### ILC2s Promote Healthy Adipose Expansion and Development

The timing and development of obesity in patients can change the outcome of T2DM and can affect its pathogenesis. For the healthy expansion of adipose tissue, there is a need for expansion of not only adipocytes but also immune cells (232). Healthy adipose expansion requires proper regulation of the endothelium in terms of adhesion molecules that enhance immune trafficking to keep up with the growing tissue as well as vascular growth factors for oxygen distribution. In unhealthy adipose tissue expansion, adipocyte hypertrophy and cellular stress lead to inflammation in the form of M1 and NK cell accumulation (56). In addition, angiogenesis decreases while fibrosis and hypoxia increase. The healthy adipose expansion increases M2 macrophages and Tregs (56). It is likely that in the case of rapid adipose expansion, endothelium adhesion molecules are unable to traffic enough ILC2s to keep up with a quickly expanding inflammatory environment. In addition, stromal cell IL-33 is unable to stimulate tissue-resident ILC2s. Sources of ILC2 activation have to expand, including adhesion molecules and stromal cell populations, with the growing adipose. Collagenous receptors such as LAIR-1 on ILC2s may also be involved in adipose expansion (233). There is likely is a lag time between when adipose is under rapid expansion and when ILC2s react. In case of extreme expansion, this lag time would lead to an unrecoverable ILC2 population. The rate at which the adipose expands would therefore have a direct effect on the immunology of the adipose and affect outcomes of T2DM in patients.

## Conclusion

ILC2s play a clear role in the pathogenesis of T2DM through the protection and maintenance of homeostasis in adipose tissues. The crosstalk between these cells, the immune environment, and cytokine milieu is crucial for the development of novel therapeutics. How different external and genetic factors play a role in affecting these cells and the homeostatic outcome of multiple tissues is a key point of interest. The delicate immunological balance between type 1 and type 2 inflammation in the adipose tissue determines outcomes of T2DM. With further understanding and research in controlling that equilibrium, more long-term treatments will be developed. Healthy systemic immunological balance is achieved through proper trafficking and maintenance of helpful immune cell populations such as ILC2s. Cell-specific agents that target these homeostasis regulating cells are going to be critical for multiple phenotypes of human T2DM. In treatments, not only is cell specificity needed, but also an understanding of the stage, timing, and immunological phenotype of each T2DM patient. With proper screening and clustering based on immunological needs, treatments for T2DM will become more efficient and provide long-term relief for patients.

## Author Contributions

JP wrote the manuscript and prepared illustrations. OA supervised and edited the manuscript. Both authors contributed to the article and approved the submitted version.

## Funding

This article was financially supported by National Institutes of Health Public Health Service grants R01 ES025786, R01 HL144790, R01 AI145813, R01 HL151493 (OA).

## Conflict of Interest

The authors declare that the research was conducted in the absence of any commercial or financial relationships that could be construed as a potential conflict of interest.

## Publisher’s Note

All claims expressed in this article are solely those of the authors and do not necessarily represent those of their affiliated organizations, or those of the publisher, the editors and the reviewers. Any product that may be evaluated in this article, or claim that may be made by its manufacturer, is not guaranteed or endorsed by the publisher.
